# Endothelial Neurogranin Regulates Blood–Brain Barrier Permeability via Modulation of the AKT Pathway

**DOI:** 10.1007/s12035-024-04522-9

**Published:** 2024-10-05

**Authors:** Adesewa O. Akande, Zachary A. Carter, Karen Y. Stokes, Hyung W. Nam

**Affiliations:** 1 Department of Pharmacology, Toxicology, and Neuroscience, Louisiana State University Health Sciences Center, Shreveport, LA 71103 USA; 2 Department of Molecular and Cellular Physiology, Louisiana State University Health Sciences Center, Shreveport, LA 71103 USA

**Keywords:** Neurogranin, Akt, Blood–brain barrier, Permeability

## Abstract

Neurogranin (Ng) expression is a biomarker for Alzheimer’s disease. A loss of brain Ng and an increase in CSF Ng positively correlate with cognitive decline. Ng is known to regulate neuronal calcium-calmodulin binding and synaptic plasticity, which are critical for learning/memory. Interestingly, we discovered that Ng is also expressed in mouse and human blood–brain barrier (BBB). However, the role of Ng expression in brain vasculature remains largely undefined. In this study, we investigated the role of Ng expression on neurovascular structure and function using Ng null mice and human cerebral microvascular endothelial (hCMEC/D3) cells. We performed brain clearing and immunolabeling of blood vessels from whole brains and brain slices. Deletion of Ng significantly decreases neurovascular density in mice. Using in vivo permeability assays, we found increased neurovascular permeability in Ng null mice. We also observed significant changes in the expression of tight junction proteins using western blot and immunofluorescent staining. To identify the molecular pathways involved, we carried out label-free proteomics on brain lysates from endothelial-specific Ng knockout mice. Ingenuity Pathway Analysis indicated that the AKT pathway is attenuated in the vasculature of endothelial-specific Ng knockout mice. To validate these in vivo findings, we pharmacologically manipulated AKT signaling in hCMEC/D3 cells and observed that inhibition of AKT activation causes increased permeability. Our results indicate that the loss of Ng expression alters neurovascular structure and permeability, potentially contributing to neurological dysfunction. Therefore, modulating Ng expression in the BBB may offer a novel therapeutic approach for Alzheimer’s disease.

**Supplementary Information:**

The online version contains supplementary material available at 10.1007/s12035-024-04522-9.

## Introduction

The blood–brain barrier (BBB) describes the unique structure and characteristics of the capillaries that vascularize the central nervous system (CNS). The BBB prevents neurotoxic plasma components, blood cells, xenobiotics, and pathogens from entering the brain parenchyma [1]. Endothelial tight junctions, lack of fenestrae, and low pinocytic/endosomal transport confer the selectivity of this barrier [2, 3]. The BBB also serves as the primary site for blood-CNS exchange [4]. In the CNS, neuronal cell bodies are within 15 µm of the closest microvessel [5]. The BBB plays an important role in regulating neuronal homeostasis, and disruption of the BBB can lead to the leakage of harmful substances into the brain. This is associated with inflammation and oxidative stress that may contribute to the pathophysiology of many neurological disorders [6] such as epilepsy [7], multiple sclerosis [8], Alzheimer’s disease [9], depression [10], and schizophrenia [11]. Since dysfunction of the BBB can also lead to cellular infiltration and aberrant transport of molecules that can affect cerebral blood flow, the BBB may be a novel target for managing neurological and psychiatric disorders.

This study investigated Neurogranin (Ng) expression in brain microvessels and its functional role in maintaining BBB integrity. Ng is a small 7.5 kDa protein that is known as a neuron-specific protein that plays an important role in long-term potentiation (LTP) in the hippocampus [12]. In neurons, Ng regulates Ca^2+^-CaM binding because it has a strong affinity for calmodulin when calcium levels are low [13, 14]. Depletion of Ng in mice causes altered calcium dynamics and deficits in cognitive function with anxiety components [15, 16]. In human clinical studies, a genetic variant of the Ng gene (Nrgn) is significantly associated with the development of schizophrenia [17–19]. In addition, Ng depletion in the brain and elevated Ng levels in the cerebrospinal fluid have been evaluated as a biomarker for Alzheimer’s disease [20, 21]. However, the underlying pathophysiological mechanism remains unknown.

Recent studies have reported the role of Ng expression in other cells outside the CNS. Ng was shown to activate Ca^2+^-CaM signaling in myoblasts [22], immune cells [23], and cardiomyocytes [24]. Moreover, several reports indicated that Ng is expressed in the vasculature, where it regulates endothelial function through Ca^2+^-CaM-mediated AKT signaling [25]. Therefore, studies need to consider the contribution of Ng expression in the BBB to brain function and behavioral outcomes, which are observed in humans and preclinical models. Among the possible downstream targets of Ng, AKT activation is involved innumerous processes such as protein synthesis, cell proliferation, cell survival, and neural plasticity [26, 27]. Interestingly, AKT activation has also been shown to be protective against BBB damage. Studies have shown that preservation of AKT activation following cerebral ischemia in mice was correlated with better BBB outcomes, such as reduced BBB permeability and increased expression of tight junction proteins [28, 29].

Thus, we explored the role of Ng expression in maintaining BBB integrity, which is critical in preventing the early progression of neurological diseases. To assess the impact of Ng expression on the BBB, we employed a transgenic mouse model, 3D mouse brain imaging, in vivo permeability, and label-free proteomics. We used in vitro BBB cell culture, trans-endothelial electrical resistance (TEER), and pharmacological manipulation to further validate the mechanism. Our findings provide a new understanding of the Ng mechanism and its specific role in BBB and neurological diseases.

## Materials and Methods

### Animals

Ng^−/−^ mice on a C57BL/6 J background were purchased from the Jackson Laboratory and backcrossed with 129S1/SvImJ mice. Male Ng^+/+^ mice and Ng^−/−^ mice (4-month-old) were used for in vivo permeability assays. Male endothelial-specific Ng knockout Cre-CDH5-Ng^f/f^ and control Cre-CDH5-Ng^+/+^ mice (C57BL/6 J background, Cyagen, 4-month-old) were used for the proteomic analysis. Mice were group-housed in standard Plexiglas cages under a 12-h light/dark cycle in temperature (24 ± 0.5 °C) and humidity-controlled rooms (60 ± 2%). Water and food were provided *ad libitum*. Animal protocols were approved by the Animal Care and Use Committee (ACUC) in the Louisiana State University Health Sciences Center Shreveport.

### Isolation of Mouse Brain Microvessel

The mice were sacrificed by placing them in an isoflurane chamber for 2–3 min and performing cervical dislocation. As described [30], mouse brains were carefully isolated and rinsed in Molecular, Cellular, and Development Biology 131 medium (MCDB 131, # 10372019 Gibco). Meninges were removed by rolling the brains on Whatman filter paper (3 M), and the olfactory bulb and cerebellum were cut off with a blade and discarded. Brain tissue was homogenized with 8 strokes of a Dounce tissue homogenizer. The homogenate was centrifuged at 2000 × g for 5 min at 4 °C, and the supernatant was discarded. The pellet was mixed with 15 mL of 15% 70 kDa dextran, vortexed carefully, and then centrifuged at 10,000 × g for 15 min at 4 °C. The supernatant was carefully discarded to prevent contamination of the pellet with the floating debris on top. The red microvessel-containing the pellet was transferred to a 40 μm cell strainer and washed with 10 mL Dulbecco’s phosphate-buffered saline (DPBS, #14040133, Thermo Fisher). The pellets were collected by reverse filtration and used for further analysis.

### Cell Culture and siRNA Treatment

Human cerebral microvascular endothelial cells (hCMEC/D3: SCC066) were purchased from Millipore Sigma. hCMEC cells were maintained using endothelial cell media (Sciencell, USA) supplemented with FBS, endothelial cell growth supplement (ECGS) and 10 U/ml penicillin, and 100 µg/mL streptomycin. Cells were grown to 60–70% confluency transfected with a siRNA directed towards Ng (20 pmol, #s9723, Thermo Fisher) using Lipofectamine RNAiMAX (#13778150, Thermo Fisher) for 6 h in Opti-MEM (#31985070, Thermo Fisher). For AKT inhibitor treatment experiments, cells were switched to media containing LY294002 (#70920, Cayman Chemical). Lipofectamine control (mock), siRNA-treated, or drug-treated samples were collected after 24 h for western blots.

### Whole-Brain Imaging and Analysis

Ng^+/+^ and Ng^−/−^ mice were transcardially perfused with ice-cold 1X PBS with 10U/mL heparin until the fluids ran clear, followed by icecold 4% PFA. The brains were extracted, and the samples were post-fixed in 4% PFA for 24 h and transferred to PBS with 0.02% sodium azide. Whole-mouse brain clearing and imaging were performed by LifeCanvas Technologies through a contracted service. As described [31], fixed whole brains were prepared with SHIELD to preserve protein before being cleared and immunolabelled with a CD31 and leptin antibody using SmartBatch + . Labeled brains were imaged by a LifeCanvas SmartSPIM Light Sheet Microscope. Images obtained from light sheet microscopy were reconstructed using the Imaris Essentials Software Package. ImageJ software was used for image processing, analysis, and quantification.

### Evans Blue Permeability Assay

Male Ng^+/+^ mice (*n* = 5) and Ng^−/−^ mice (*n* = 6) aged 8–12 weeks were used in this assay. 200 µL of 2 mg/mL Evans Blue dye was injected into the tail veins of the mice. The dye was circulated for 30 min, and then the mice were perfused with ice-cold PBS for 10 min. Their brains, hearts, and lungs were collected. The collected organs were homogenized in formamide and centrifuged; then the absorbance of the supernatant was measured at 620 nm. The concentration of Evans blue per mg tissue in the brain samples was then determined from a standard curve.

### Dextran Permeability Assay

Using the stereotactic surgical apparatus (KOPF Instruments), 1 µL of 2 mg/mL 40 kDa fluorescein (FITC) dextran (FD40S, Sigma Millipore) was injected bilaterally into the ventricles (stereotactic coordinates ± 1.0 mm ML, − 0.3 mm AP, and − 2.5 mm DV from bregma). The dextran was allowed to exit the ventricles into the blood for 2 h. 500 µL of whole blood was collected from the inferior vena cava and allowed to sit on dry ice in the dark for 1 h. The whole blood was centrifuged at 2000 g for 10 min to separate serum from cellular components, and 100 µL of the supernatant serum was collected for analysis. Fluorescent intensity was measured at the excitation and emission wavelengths of 488 nm and 528 nm, respectively. The amount of 40 kDa FITC dextran in the samples was determined from a standard curve and adjusted for the estimated total blood volume.

### Western Blotting

Bilateral 1.5 mm tissue punch samples from the medial prefrontal cortex (mPFC) of Ng^+/+^ and Ng^−/−^ mice were collected and homogenized in 1X RIPA lysis buffer (#20188, Millipore) with protease/phosphatase inhibitor cocktail (#78442, Thermo Fisher). Homogenates were centrifuged, and supernatants were collected for western blotting. For the cell lysates, samples were collected 24 h post-transfection or post-drug treatment. The cells were lysed in M-PER (#78505, Thermo Fisher) buffer with protease/phosphatase inhibitor cocktail (#78442, Thermo Fisher). Proteins were separated with 4–20% PROTEAN TGX gels for 1 h at 120 V. The gels were transferred to activated PVDF membranes using the Trans-Blot Turbo Transfer System (#1704150, Biorad) protocol for mixed molecular weight proteins. Membranes were blocked with 5% milk and incubated with primary antibodies in 2% milk at 4 °C overnight. The next day, the membranes were washed with TBST and incubated with appropriate HRP-linked secondary antibodies. Chemiluminescent bands were detected on a Bio-Rad imaging station and quantified using the ImageJ software.

### Immunocytochemistry

For immunofluorescence staining of hCMEC/D3 cells, mock controls and Ng-siRNA-treated cells were grown to 70% confluency on coverslips in 24-well plates and fixed with 2% paraformaldehyde. Fixed cells were permeabilized with 0.2% Triton X-100 in PBS for 10 min and blocked with 1% BSA and 0.1% azide for 1 h at room temperature. The cells were incubated with primary antibodies for neurogranin, CD31 overnight at 4 °C. Following this, the cells were incubated with appropriate Alexa Fluor conjugated secondary antibodies; the coverslips were washed three times with TBST for 10 min each and mounted with Prolong™ Diamond Antifade Mountant with DAPI (#P36971, Invitrogen). After curing for 24 h, images were acquired using the Nikon A1R Confocal & Super Resolution System.

### Transendothelial Electrical Resistance (TEER) Measurement

After transfection, the cells were digested with trypsin and plated on rat-tail collagen-coated cell culture polycarbonate transwell inserts (#3413, Corning) in the TEER24 gold electroarray plates (Applied biosciences) at a density of 10^5^ per mL and grown endothelial cell media supplemented with FBS, ECGS and 10 U/ml penicillin, and 100 µg/mL streptomycin. Four wells were left without cells for zeroing and electrode drift correction. TEER was measured continually for 72 h for the mock and Ng-siRNA groups using an electric cell-substrate impedance sensing (ECIS) station (Applied Biophysics). For the drug treatment, cells were switched to media containing 10 µM LY294002 72 h after the cells were transferred to the trans wells, and TEER was measured for 30 h post-drug treatment.

### Brain Tissue Sample Preparation for Proteomic Analysis

Ng^f/f^ mice (*n* = 4) and Cre-CDH5-Ng^f/f^ mice (*n* = 5) were subjected to isoflurane gas to induce unconsciousness, followed by PBS perfusion and isolation of the mPFC. The extracted tissue was snap-frozen on dry ice and stored at − 80 °C until it was processed for SDS-PAGE (Bio-Rad Criterion system). Each replicate was homogenized in an extraction buffer containing 50 mM Tris (pH 7.4), 2 mM EDTA, 5 mM EGTA, and 0.1% SDS protease inhibitor cocktail type I (Roche) and II (Sigma). Homogenates were centrifuged at 500 g at 4 °C, and supernatants were collected. Protein concentration from each replicate supernatant was quantified using the Bradford protein assay (#5,000,202 BioRad). Samples were loaded at 30 µg and separated via electrophoresis in a 4–12% polyacrylamide gel, followed by sample preparation for proteomic analysis.

### Label-Free Proteomics

1D gels were divided into 6 sections using a Precision Plus Kaleidoscope standards (#1610375, Bio-Rad) ladder. The gel fractions were dehydrated and alkylated using 100% acetonitrile and 40 mM iodoacetamide (#407710, Millipore Sigma) in 50 mM Tris for 1 h at room temperature. Samples were rehydrated in 25 mM Tris and subjected to trypsin digest. Each gel piece was subjected to in-gel trypsin digestion as follows. Gel segments were destained in 50% methanol and 50 mM ammonium bicarbonate, followed by a reduction in 10 mM Tris and alkylation in 50 mM iodoacetamide. The gel slices were then dehydrated in acetonitrile, followed by adding 100 ng porcine sequencing grade modified trypsin in 50 mM ammonium bicarbonate and incubating at 37 °C for 16 h. Peptide products were then acidified in 0.1% formic acid. Tryptic peptides were separated by reverse-phase XSelect CSH C18 2.5 µm resin on an in-line 150 × 0.075 mm column using a nanocavity UPLC system. Peptides were eluted using a 60-min gradient. Eluted peptides were ionized by electrospray (2.4 kV) followed by MS/MS analysis using higher-energy collisional dissociation (HCD) on an Orbitrap Fusion Tribrid mass spectrometer in top-speed data-dependent mode. MS data were acquired using the FTMS analyzer in profile mode at a resolution of 240,000 over 375 to 1500 m/z. Following HCD activation, MS/MS data were obtained using the ion trap analyzer in centroid mode and normal mass range with precursor mass-dependent normalized collision energy between 28.0 and 31.0.

### Label-Free Quantification Data Analysis

Proteins were identified by database search using Mascot Distiller (version 2.5.1, Matrix Science) with a parent ion tolerance of 3 ppm and a daughter ion tolerance of 0.5 Da. All MS/MS samples were analyzed using Mascot (Matrix Science, London, UK; version 2.6.2). The mascot was set up to search the UniProt_2022_database, assuming the digestion enzyme trypsin. Mascot was searched with a fragment ion mass tolerance of 0.5 Da and a parent ion tolerance of 3.0 ppm. Carbamidomethyl of cysteine was specified in Mascot as a fixed modification. Oxidation of methionine and acetyl of the N-terminus were specified in Mascot as variable modifications. A maximum of two missed cleavages were allowed. The minimum number of peptides for positive protein identification was set to at least one unique peptide sequence. Protein identifications were confirmed by duplicated sample analysis. Mascot search results (DAT files) were imported into Scaffold to verify MS/MS-based peptide and protein identifications. Scaffold (version Scaffold_5.1.2, Proteome Software Inc., Portland, OR) was used to validate all samples’ MS/MS-based peptide and protein identifications. Peptide identifications were accepted if they could be established at greater than 99.0% probability to achieve an FDR less than 1.0% by the Peptide Prophet algorithm [32] with Scaffold delta-mass correction. Protein identifications were accepted if they could be established at a probability greater than 99.0% to achieve an FDR of less than 1.0% and contained at least 2 unique peptide sequences. Proteins that contained similar peptides and could not be differentiated based on MS/MS analysis alone were grouped to satisfy the principles of parsimony. Detection of differentially expressed peak ratios was performed with ANOVA utilizing a signature *p*-value decoy error rate of no more than 5%, and this annotation was assigned across aligned sample features so that relative quantitation of corresponding protein identifications across sample groups could be compared [32–34].

### Ingenuity Pathway Analysis (IPA) for Protein Classification

We used Ingenuity Pathway Analysis (IPA) to identify the functional pathways of the proteins with altered expression. We entered the genes that had fold changes greater than 1.2-fold, and *p* values less than 0.05 into IPA for the core analysis. IPA shows potential networks involved in microarray or proteomic profiles by the IPA Network Generation Algorithm. Proteins were clustered and classified by the IPA Network generation algorithm, and the network score ranked the networks. Solid and dashed lines indicate direct and indirect network interactions, respectively. Direct interactions require that the two molecules make direct physical contact without intermediate steps. Indirect interactions do not need a physical connection between the two molecules, such as interactions in a signaling cascade.

### Heat Map Analysis and Principal Component Analysis

Heat maps were drawn to determine the expression patterns of significantly up-regulated or down-regulated protein at each time point and to compare the expression levels with the other time points, using R version 2.15.1 and the R packages. PCA can reduce the dimensionality of a data set consisting of many interrelated variables while retaining the variation in the data set as much as possible. PCA was conducted as an “unsupervised” analysis to clarify the variance among microarray data from the brain samples using R. To clarify the variances among samples, data were calculated using a Q-mode PCA package ‘prcomp function.’ The proportion of variance and factor loading were also calculated.

### Experimental Design and Statistical Analysis

We employed the in vitro hCMEC/D3 cell culture model and in vivo Ng knockout (Ng^−/−^) mouse model to study brain vascular permeability and BBB integrity. To determine the role of Ng expression in BBB permeability, we established the gene transfection models in the hCMEC/D3 cell line for Ng knockdown using the Ng-siRNA approach. This gene expression method was compared with three different gene constructs and two different concentration conditions and finally optimized to the best condition. Lipofectamine treatment without the Ng construct was used as a mock control. We first measured 3D whole-brain vasculature using brain clearing and Light Sheet Fluorescent Microscopy to determine brain vascular structural changes in response to Ng knockout. Then, we selected the mPFC brain region as a target brain and conducted immunofluorescence for CD31 staining after brain tissue clearing using confocal microscopy. To identify the molecular signaling changes in the brain of endothelial-specific Ng knockout mice, label-free proteomics using nanoLC-ESI–MS/MS experiments was employed. Each sample run was duplicated. Label-free proteomics was achieved using MS/MS spectra counts using Scaffold 5 software. Ingenuity Pathway Analysis (IPA) bioinformatic analysis elucidated relevant pathways related to endothelial-specific Ng knockout. Then, the target pathways were validated using western blotting (*n* = 3). The data are shown as the mean ± standard error of the mean (SEM). To detect statistical differences, we performed a two-tailed Student’s *t*-test or an analysis of variance (ANOVA) where appropriate (Prism, GraphPad Software, La Jolla, CA). The criterion for statistical significance was *p* < 0.05.

## Results

### Neurogranin Is Expressed in Brain Microvessels in Both Humans and Mice

**Fig. 1:**
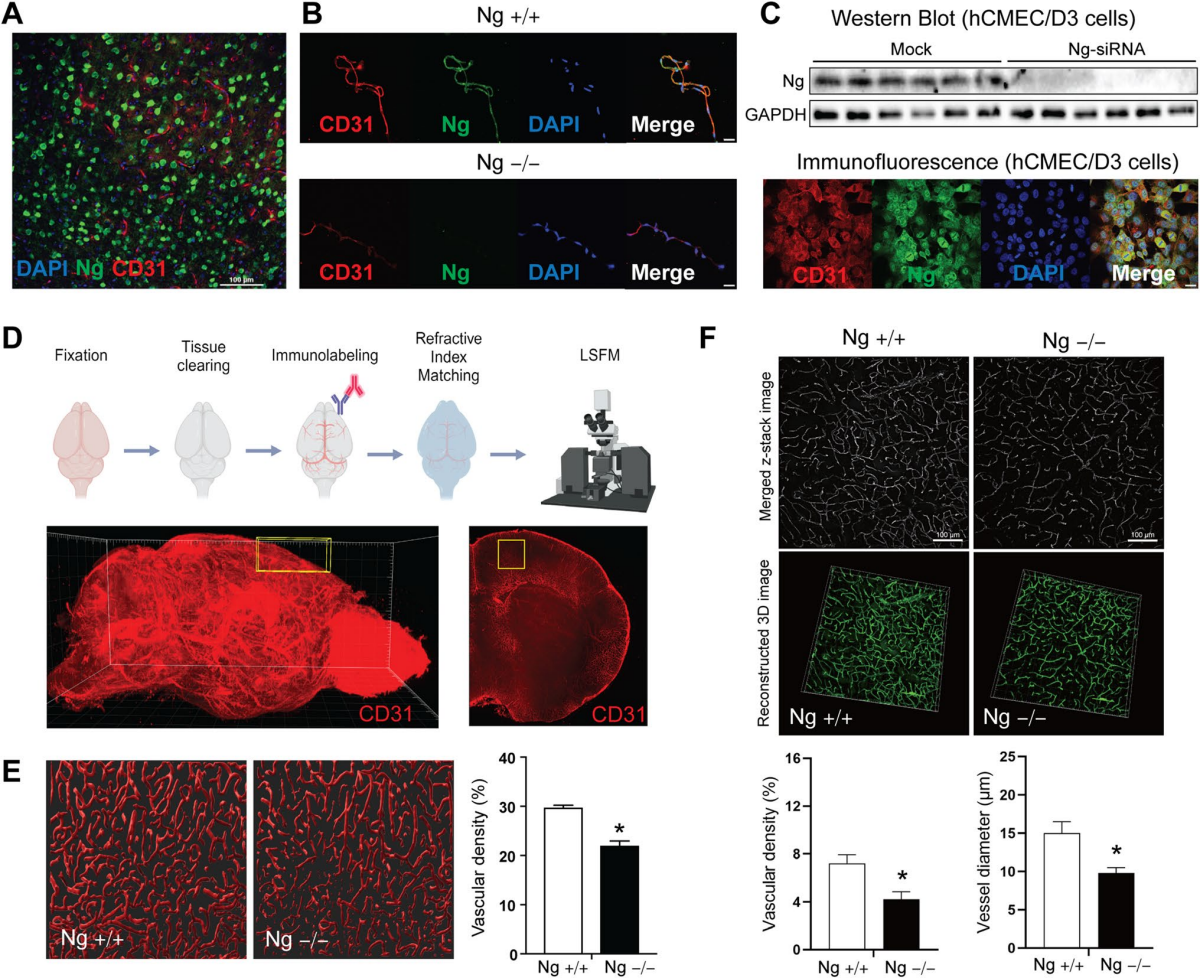
Neurogranin is expressed in the brain microvessel in both humans and mice. **A** Immunofluorescence (IF) staining of cortical brain sections from Ng^+/+^ mice. Co-staining of Ng (green), CD31 (red), and DAPI (blue) show strong neuronal expression of Ng. **B** IF analysis of extracted microvessels from Ng^+/+^ and Ng^−/−^ mice using co-staining of Ng (green), CD31 (red), and DAPI (blue). Ng^+/+^ mice show colocalization of Ng expression with CD31, while there is no Ng expression in the brain microvessels from Ng^−/−^ mice (scale bar, 20 µm). **C** Representative immunoblot staining for Ng of lysates of hCMEC/D3 cells. Ng knockdown was achieved with Ng-siRNA in hCMEC/D3 cells. IF staining of Ng (green), CD31 (red), and DAPI (blue) of fixed hCMEC/D3 cells (scale bar 20 µm) reveals the expression of Ng in hCMEC/D3 cells. **D** Schematic for the whole-mouse brain clearing and immunolabelling of blood vessels from Ng^+/+^ and Ng^−/−^ mice. Whole brains were fixed with PFA and cleared with SHIELD protocol. CD31 immunolabelling was imaged with a light sheet fluorescent microscope (LSFM). 3D-rendered image of whole brain blood vessels (stained with CD31) from a Ng^+/+^ mouse. The yellow box shows the region of interest (ROI; mPFC) used to quantify changes in blood vessel density (1 mm × 1 mm × 2.4 mm = 2.4 mm^3^). **E** Representative images of 3D rendered blood vessels from the mPFC of Ng^+/+^ and Ng^−/−^ mice (thickness = 40 μm) . Significantly decreased vascular density was observed in the Ng^−/−^ mice (*n* = 6 per mouse brain). **F** 3D reconstructed images of IF CD31 stained brain sections from Ng^+/+^ and Ng^−/−^ mice (scale bar, 100 µm. Images were acquired with a Nikon confocal microscope (thickness = 80 µM) after a SHIELD-based staining approach. Decreased vascular density and diameter were observed in the mPFC of Ng^−/−^ mice (*n* = 4 per genotype)). Vascular density and vascular diameter were quantified using the ImageJ vessel analysis plugin. Data are mean ± SEM. *p < 0.05; two-tailed unpaired Student’s *t*-test

Although Ng is known as a neuron-specific protein, we found that Ng expression is also expressed in the blood vessels of wild-type mouse brains. Cortical brain tissues of wild-type (Ng^+/+^) mice were stained with Ng and CD31, the endothelial cell marker (Fig. 1A). There were no areas of colocalization between Ng (green) and CD31 (red) due to a substantial amount of neuronal Ng expression in cortical area. Therefore, we extracted microvessels from both Ng^+/+^ and Ng^−/−^ mice and carried out immunofluorescence (IF) staining for CD31 and Ng. The colocalization results from the extracted microvessel of the Ng^+/+^ mice indicate that Ng protein is co-localized with CD31, while there is no Ng expression in the microvessels from Ng^−/−^ mice (Fig. 1B). Thus, to confirm Ng expression in brain microvessel, we assessed Ng protein expression in the human cerebral microvascular endothelial cells/D3 (hCMEC/D3). Using Western blotting, we identified the Ng protein in the hCMEC/D3 and validated its expression using Ng-siRNA (Fig. 1C). Immunofluorescence results indicate that Ng (green) is expressed in the hCMEC/D3 and co-localized with CD31 (red) (Fig. 1C). These in vivo and in vitro results confirm that Ng protein is expressed in mice and humans’ blood–brain barrier (BBB).

Then, we evaluated how the depletion of Ng impacts brain vasculature using 3D whole-brain imaging approaches. The whole brain of each genotype was cleared and stained with CD31 and imaged with Laser Sheet Fluorescence Microscopy (LSFM) (Fig. 1D). Individual LSFM images were processed and reconstructed into 3D images using Imaris image analysis software. The medial prefrontal cortex (mPFC) was selected as the region of interest, highlighted by the yellow box (1 mm × 1 mm × 2.4 mm: 2.4 mm^3^). We analyzed the vascular density of 40 µm of 3D rendered mPFC tissues between genotypes using CD31, and we identified a significant decrease in vascular density in the mPFC Ng^−/−^ mice compared to Ng^+/+^ mice (*n* = 6 cortical layers per brain, *t* = 6.908, df = 10, *p* < 0.0001) (Fig. 1E). To further validate the LSFM-based 3D rendering, we applied the same immunofluorescent approaches by immunolabelling cleared mouse cortical sections. (80 µm thick) with CD31 using a super-resolution microscope (*n* = 4 per genotype). Then, we reconstructed CD31-stained blood vessels by *Z*-stacking individual images. In the mPFC of Ng^−/−^ mice, vessel analysis revealed significantly decreased vessel density and vessel diameter compared to wild-type mice (Fig. 1F). This data indicates that the depletion of Ng impacts brain vascularization, which may impact BBB function and integrity.

### Neurogranin Depletion in Mice Increases BBB Permeability

**Fig. 2:**
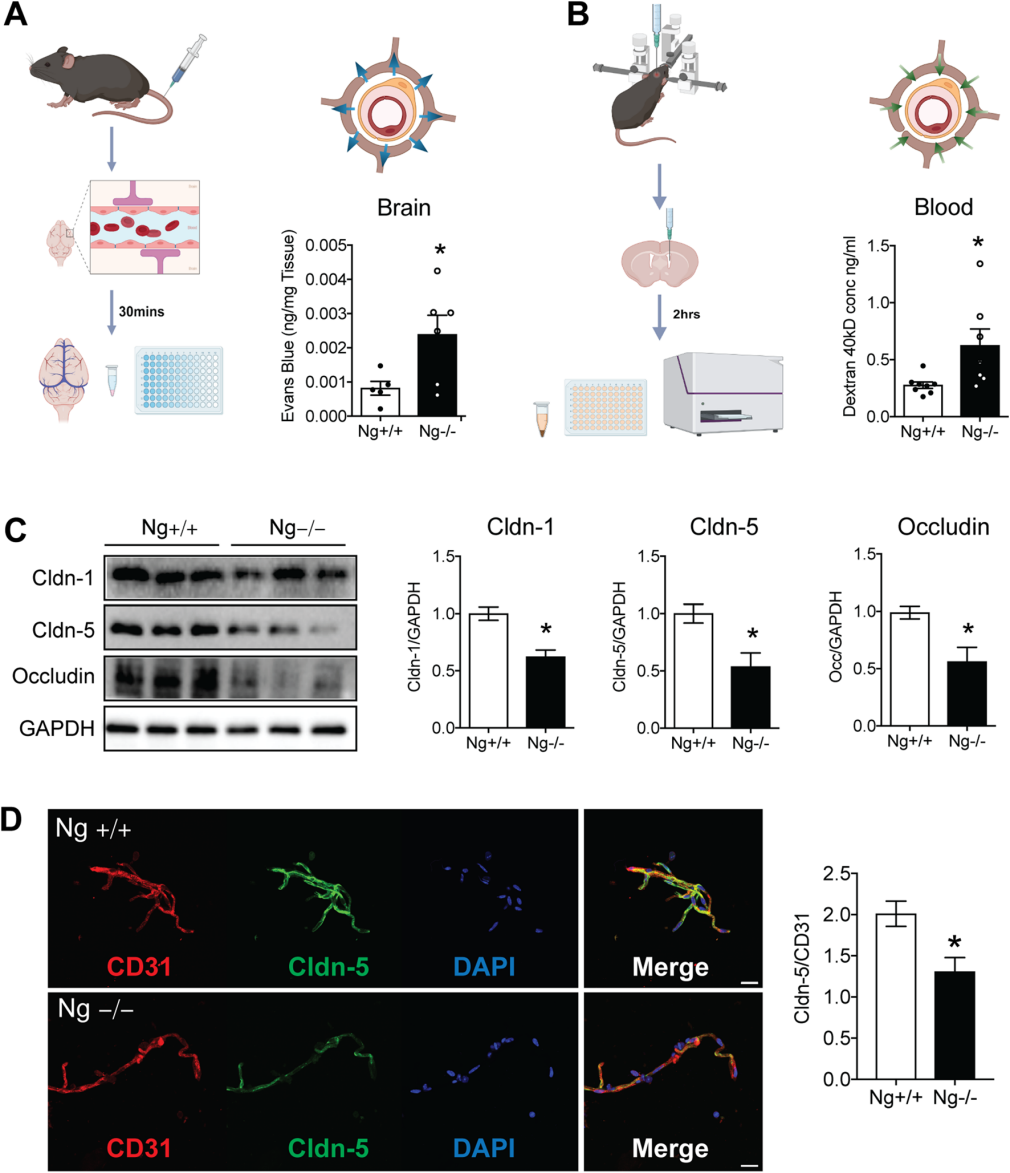
Increased apical and basolateral BBB permeability in Ng^−/−^ mice. **A** Schematic for the apical Evans blue permeability assay. Evans blue was injected into the tail veins of Ng^+/+^ mice and Ng^−/−^ mice. Then, brains were collected after 30 min, and the amount of Evans blue dye in the brain parenchyma was quantified. Quantification of Evans blue in the brain parenchyma in ng/mg of tissue showed significantly increased Evans blue dye in the brains of Ng^−/−^ mice. **B** Procedure for the Dextran permeability assay. Ng^+/+^ and Ng^−/−^ mice were given intraventricular injections of 40 kDa FITC dextran, and blood was collected 2 h later. The amount of blood 40 kDa FITC dextran was measured. Ng^−/−^ mice had increased blood levels of 40 kDa FITC dextran. **C** Western blot analysis of lysates from the brain of Ng^+/+^ and Ng^−/−^ mice. Immunoblots show a decrease in claudin-1, claudin-5, and occludin expression. Protein expression changes were normalized to GAPDH. **D** Representative IF images from mPFC microvessels from Ng^+/+^ and Ng^−/−^ mice, claudin-5 (green) CD31 (red), and DAPI (blue) (scale bar, 20 µm). Quantification of claudin-5 expression, showed a decrease in claudin-5 fluorescence in microvessels from Ng^−/−^ mice. Data are mean ± SEM. *p < 0.05. Two-tailed unpaired Student’s *t*-test

To assess the effect of lack of Ng expression on BBB function, we measured in vivo BBB permeability using Ng knockout (Ng^−/−^) mice. Evans blue dye was utilized to measure the apical-basolateral permeability of both Ng^+/+^ mice and Ng^−/−^ mice. Since Evans blue is a small dye that binds to 60 kDa serum albumin, we isolated the brain to measure the crossing of blood Evans blue dye across the BBB. The Ng^−/−^ mice showed significantly increased extravasation of Evans blue into the brain parenchyma (*n* = 5–6, *t* = 2.415, df = 9, *p* = 0.0389) (Fig. 2A). There were no significant vascular permeability changes in the lungs or hearts in response to the loss of Ng expression. To assess basolateral-apical permeability, 40 kDa FITC dextran was stereotactically injected into the ventricles of Ng^+/+^ and Ng^−/−^ mice. After 2 h, blood samples were collected from both genotypes. Then, serum concentrations of FITC dextran were quantified. Ng^−/−^ mice had significantly increased blood FITC dextran concentrations (*n* = 7–8, *t* = 2.517, df = 13, *p* = 0.0258) (Fig. 2B). These results indicate that the lack of Ng in the BBB causes increased BBB permeability. Since we observed both apical and basolateral transport changes in Ng^−/−^ mice, we anticipated that Ng expression plays an important role in the permeability of change through paracellular transport rather than transcellular transport, which uses intracellular transport mechanisms through the membrane. To evaluate the effect of Ng expression on paracellular transport, we quantified tight junction protein expression using western blots. mPFC lysates from Ng^−/−^ mice showed significantly decreased expressions of tight junction proteins claudin-1 (*n* = 4–5, *t* = 4.476, df = 7, *p* = 0.0029), claudin-5 (*n* = 3, *t* = 3.191, df = 4, *p* = 0.0332), and occludin (*n* = 3, *t* = 3.130, df = 4, *p* = 0.0352) compared to wild-type (Fig. 2C) indicating decreased BBB integrity. Then, cerebral microvessels from both Ng^+/+^ mice and Ng^−/−^ mice were isolated to validate the change in claudin-5 expression using immunofluorescence (Fig. 2D). The results indicated that Ng^−/−^ mice showed a significantly decreased claudin-5/CD31 ratio (*n* = 3, *t* = 2.961, df = 26, *p* = 0.0065), suggesting decreased tight-junction protein expression in the BBB.

### Lack of Ng Decreased Tight Junction Protein Expression in hCMEC/D3 Cells

**Fig. 3:**
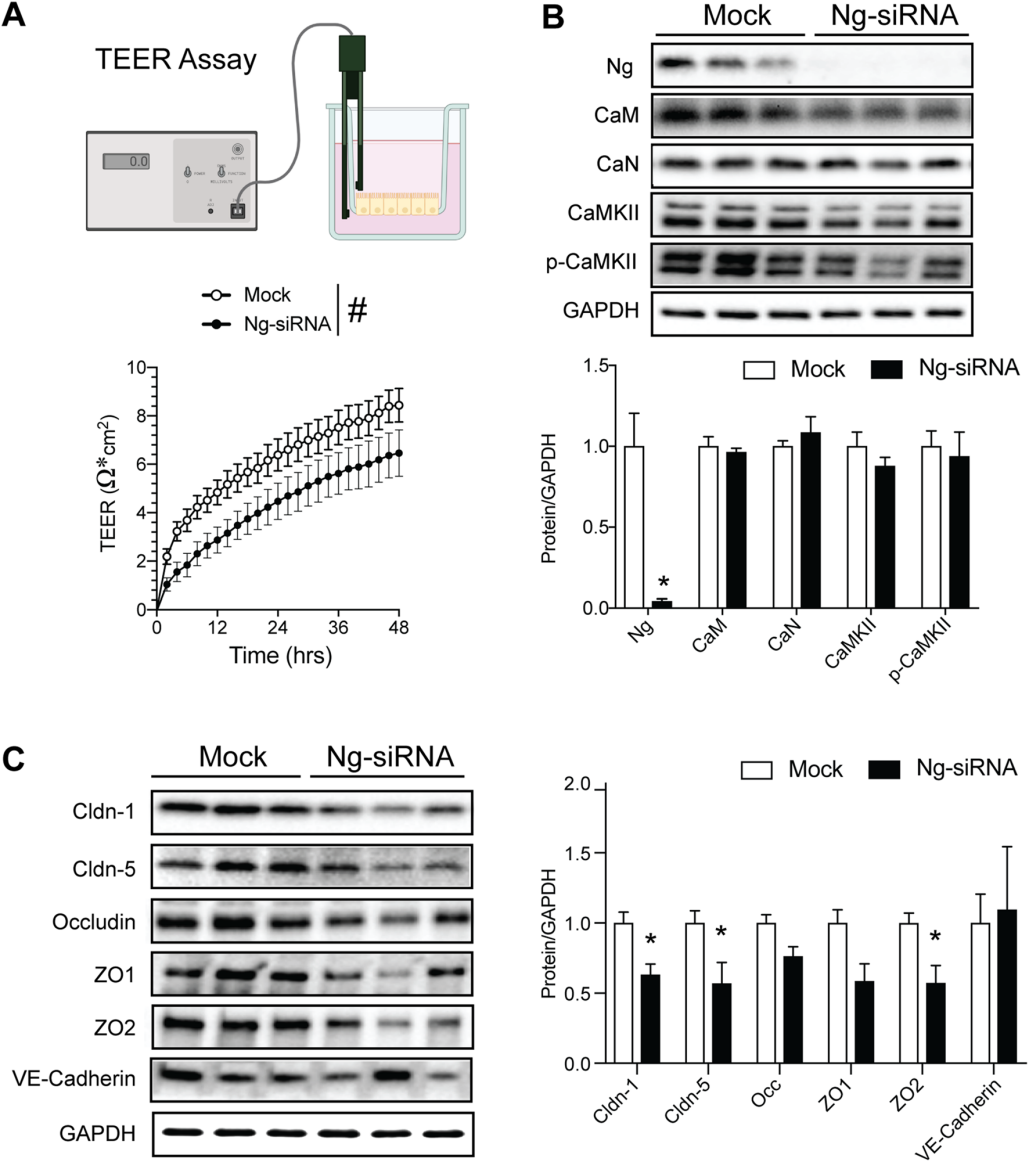
Decreased Ng expression increases permeability in hCMEC/D3 cells. **A** Methodology for the TEER assay. Continuous TEER was measured over a monolayer of hCMEC/D3 cells cultured on transwell filters. The line graph shows the development of TEER of lipofectamine control (Mock) cells versus cells treated with Ng-siRNA, indicating a sustained decrease in TEER in Ng-siRNA treated cells. ^#^*p* < 0.05; 2-way ANOVA, *n* = 7 per group. **B** Protein expression of neurogranin (Ng), calmodulin (CaM), calcineurin (CaN), pan-CAMKII, and phospho-CAMKII (p-CaMKII) in mock and Ng-siRNA-treated hCMEC/D3 cells. There were no significant changes in the expression of CaM, CaN, CaMKII, and p-CaMKII following Ng-siRNA treatment in hCMEC/D3 cells **C** Protein expression change of claudin-1, claudin-5, occludin, zonula occludens-1 (ZO1), zonula occludens-2 (ZO2), and VE-Cadherin between mock and Ng-siRNA-treated hCMEC/D3 cells. Claudin-1, claudin-5, and zonula occludens-2 (ZO2) showed significantly reduced protein expression, while there were no changes in occludin, zonula occludens-1, and VE-cadherin expression (*n* = 3). Data are mean ± SEM. *p < 0.05. Two-tailed unpaired Student’s *t*-test

Our in vivo results indicated that the depletion of Ng in the BBB decreases tight junction protein and increases paracellular permeability. To recapitulate these findings, we employed in vitro human cerebral microvascular endothelial cells (hCMEC/D3) with the Ng-siRNA model. We knocked down Ng expression using Ng-siRNA and measured the in vitro permeability using a transendothelial resistance (TEER) assay. We measured transcellular resistance after siRNA treatment in a CO_2_ incubator. Results indicated that Ng-siRNA-treated cells showed decreased resistance over 48 h. The 2-way ANOVA revealed a significant decrease of TEER in the Ng-siRNA-treated cells compared to mock in response to time [*F*(24,360) = 82.21, *p* < 0.0001] and genotype [*F*(1,15) = 4.59, *p* < 0.048], but without interaction [*F*(24,360) = 0.87, *p* = 0.64] (Fig. 3A). This TEER finding also supports that lack of Ng has a detrimental effect on BBB integrity, as observed in our in vivo Evans blue and FITC dextran assays.

Neuronal Ng expression regulates Ca^2+^-CaM-dependent signaling pathways, including Ca^2+^-CaM-dependent protein kinase II (CaMKII) and calcineurin (CaN). We measured CaM, CaMKII, and CaN protein expression in the mock (lipofectamine control) and Ng-siRNA-treated hCMEC/D3 cells to determine the mechanism of Ng-mediated permeability change. Notably, there were no significant changes in Ca^2+^-CaM-dependent signaling pathways in hCMEC/D3 cells (Fig. 3B). Furthermore, we compared tight junction protein expression between mock and Ng-siRNA. The Ng-siRNA-treated cells showed significantly less expression of claudin-1 (*n* = 3, *t* = 3.44, df = 4, *p* = 0.02), claudin-5 (*n* = 3, *t* = 2.59, df = 4, *p* < 0.05), and zonula occludens-2 (ZO2) (*n* = 3, *t* = 2.99, df = 4, *p* = 0.04), while there are no changes in occludin, zonula occludens-1 (ZO1), and VE-cadherin (Fig. 3C, D). These decreases in junction protein expression from in vitro experiments are consistent with our in vivo findings, but the molecular mechanism by which Ng expression regulates permeability changes remains unclear.

### Large-Scale Label-Free Proteomic Analysis of the mPFC of Cre-CDH5-Ng^f/f^ Mice

**Fig. 4:**
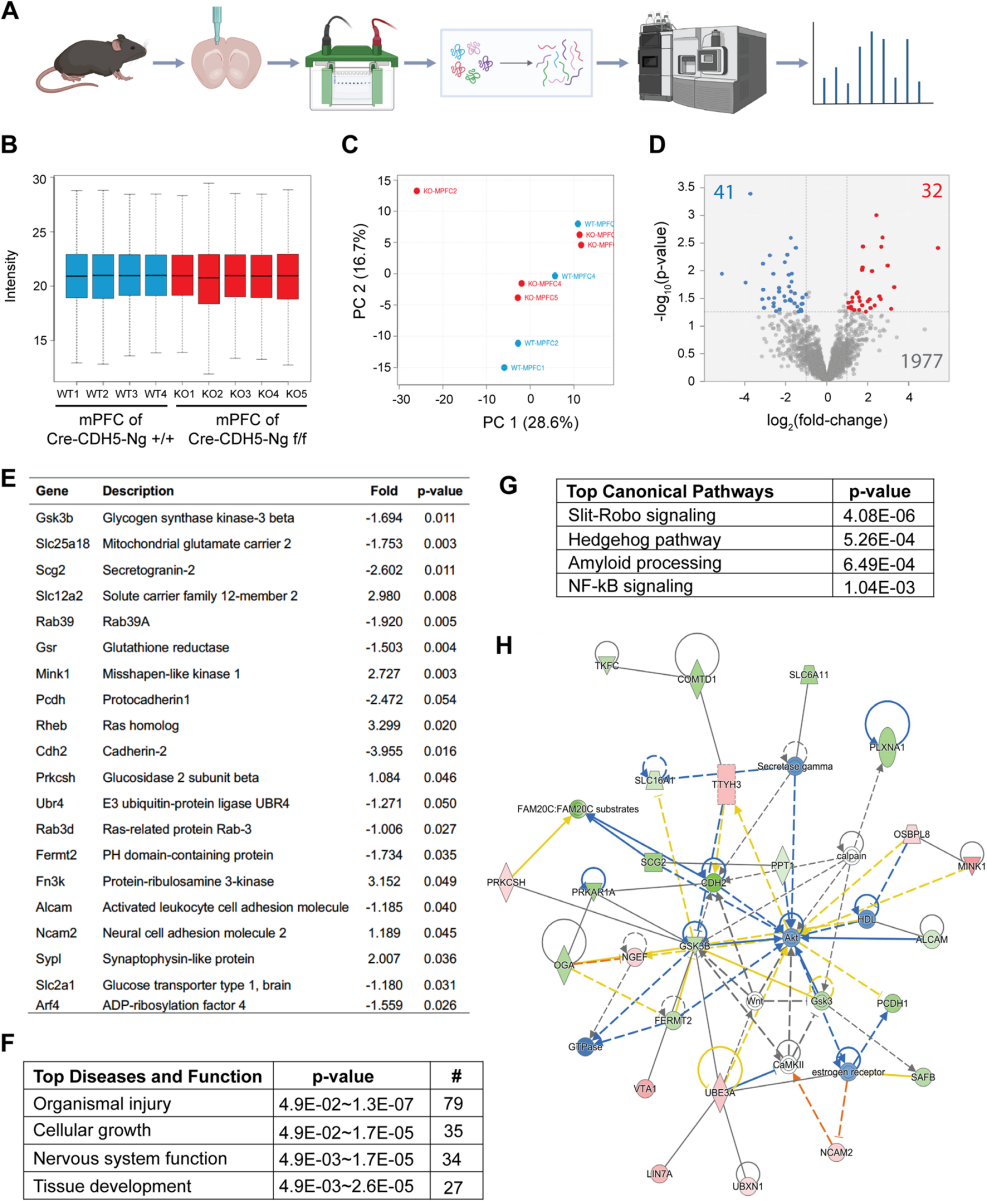
Proteomic analysis of cortical lysates from Cre-CDH5-Ng^f/f^ mice. **A** Schematic for the label-free proteomic analysis of lysates from the mPFC of Cre-CDH5-Ng^+/+^ and Cre-CDH5-Ng^f/f^ mice. **B** Box plots of MS peak intensity and peptide. The samples showed less than 10% variability. **C** Principal component analysis of proteome from the mPFC of Cre-CDH5-Ng^+/+^ (blue) and Cre-CDH5-Ng^f/f^ (red) mice. No significant differences in PCA were observed in either PC1 or PC2. **D** Volcano plot of the expression of all the proteins quantified via MS/MS. 73 proteins with significantly altered expression are highlighted in red (32 increased expression in Cre-CDH5-Ng^f/f^) and blue (41 decreased expression in Cre-CDH5-Ng^f/f^). **E** A summary of 20 selected proteins that had significantly altered expression in Cre-CDH5-Ng^f/f^ mice, showing the fold change and *p*-value. **F** Ingenuity pathway analysis (IPA) results for top diseases and functions regulated by the proteins with significantly altered expression in Cre-CDH5-Ng^f/f^ mice. **G** IPA results for the top canonical pathways regulated by the proteins with significantly altered expression in Cre-CDH5-Ng^f/f^ mice. **H** The top molecular pathway associated with the protein expression change in Cre-CDH5-Ng^f/f^ mice brain was the AKT pathway. The predicted decrease in the AKT signaling pathway was a strong candidate for further analysis

To determine the mechanism underlying Ng-mediated changes in the permeability of the BBB, we carried out label-free quantification proteomics using endothelial-specific Ng knock-out (Cre-CDH5-Ng^f/f^) mouse brain. 1 mm^3^ of mPFC tissues were isolated using micro-punches. Then, proteins were separated by 1D-SDS PAGE and analyzed using LC–MS/MS-based-label free quantification proteomics (Fig. 4A). To validate the quality of the MS/MS data from four Cre-CDH5-Ng^+/+^ mice and five Cre-CDH5-Ng^f/f^ mice, MS peak intensity and peptide number from each mouse sample were analyzed and represented as a box plots to characterize the distribution of values. All the samples showed less than 10% variability in peptide number and peak intensity (Fig. 4B). Then, a principal component analysis (PCA) was performed to analyze the similarities between Cre-CDH5-Ng^+/+^ mice and Cre-CDH5-Ng^f/f^ mice. Both genotypes did not show discriminated data patterns by PC1 or PC2, indicating a moderate impact of protein expression by endothelial-specific Ng knock-out in the mPFC tissue (Fig. 4C).

Using MS/MS-based peptide sequencing, 1977 proteins were identified in the mPFC of Cre-CDH5-Ng^+/+^ mice and Cre-CDH5-Ng^f/f^ mice. Among the 1977 cortical proteins identified, 73 proteins reached the cutoff > 1.2 of log_2_ fold change and < 0.5 log *p*-value. A volcano plot of the log_2_ fold change vs the log p-value revealed that 41 proteins were downregulated, and 32 proteins were upregulated in Cre-CDH5-Ng^f/f^ mice compared to Cre-CDH5-Ng^+/+^ mice (Fig. 4D and Supplemental Table 1). Among 73 altered proteins in Cre-CDH5-Ng^f/f^ mice, 20 proteins with significantly altered protein expression, including GSK3β, neural cell adhesion molecule 2, and cadherin-3, are highlighted (Fig. 4E). To gain further insight into the unique pathways that are changed by a loss of Ng expression in the BBB, we used the ingenuity pathway analysis (IPA). The top diseases and functions suggested by IPA are organismal injury and cellular growth, which are relevant to BBB dysfunction (Fig. 4F). Top canonical pathways suggested Slit-Robo signaling, which regulates cell motility, contributing to neuronal migration and angiogenesis (Fig. 4G). Finally, IPA molecular network analysis indicated that AKT could significantly impact how the loss of Ng expression affects endothelial cells (Fig. 4H). Based on this finding, we hypothesized that a loss of endothelial Ng expression caused an attenuation of AKT signaling that may increase BBB permeability.

### Neurogranin Expression Regulates the AKT Pathway in the BBB

**Fig. 5:**
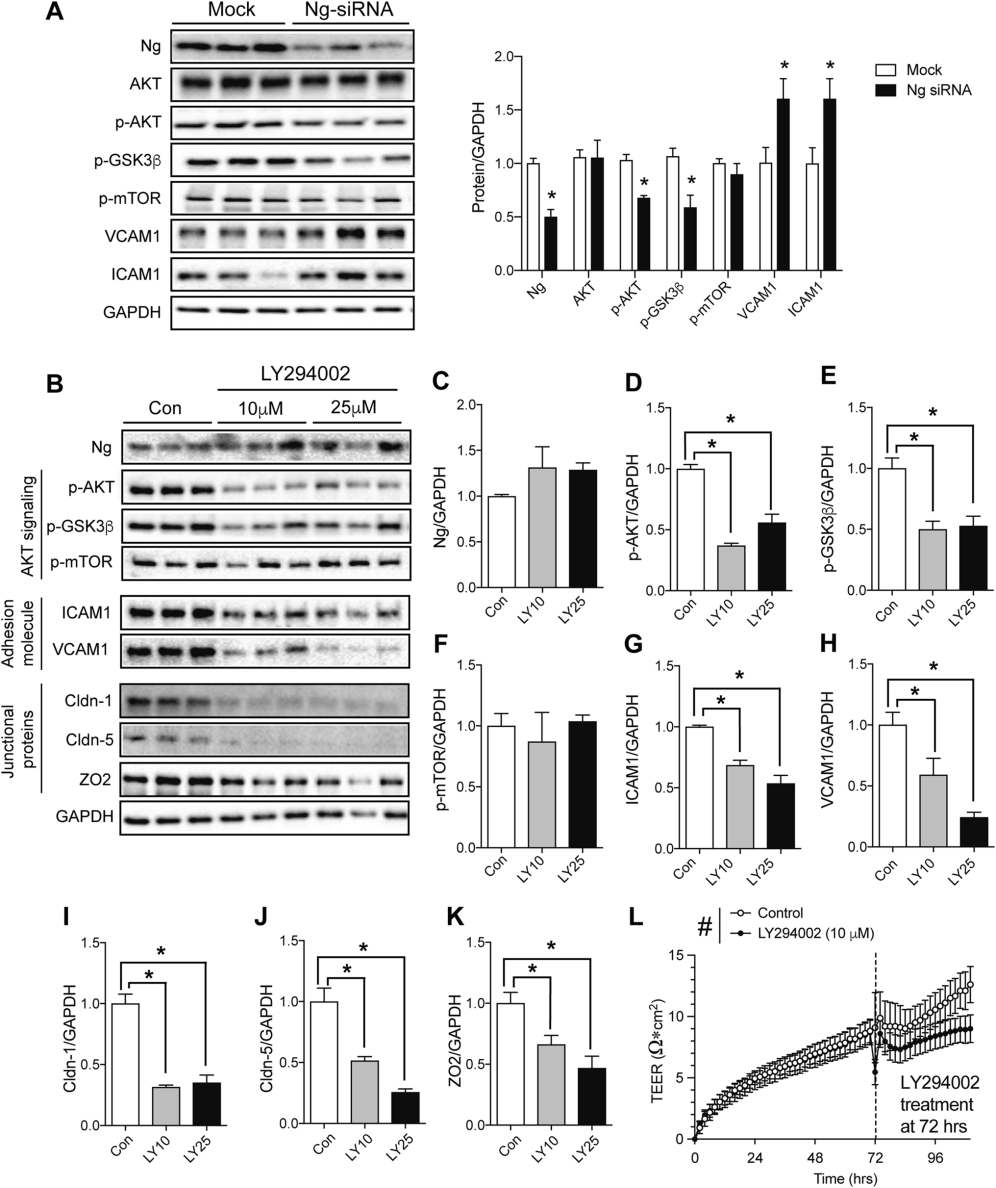
Effects of Ng knockdown on AKT signaling in hCMEC/D3 cells. **A** Protein expression changes in the AKT pathway. Ng, AKT, p-AKT, p-GSK3β, p-mTOR, VCAM1, and ICAM expression in mock and Ng-siRNA-treated hCMEC/D3 cells. Ng-siRNA significantly decreased the expression of p-AKT and p-GSK3β and increased the expression of VCAM1 and ICAM1 in hCMEC/D3 cells. p-mTOR expression remained unchanged. Protein expression changes were normalized to GAPDH (*n* = 3). **B** Representative immunoblots showing the effect of LY294002 treatment on AKT signaling, adhesion, and tight junction proteins in hCMEC/D3. **C** Ng expression in hCMEC/D3 cells remained unchanged following AKT inhibitor, LY294002 (10 µM and 25 µM) treatment. **D–F** LY294002 (10 µM and 25 µM) treatment significantly decreased p-AKT and p-GSK3β expression, while there was no effect on p-mTOR expression. **G, H** LY294002 treatment significantly decreased the expression of cell adhesion molecules ICAM1 and VCAM1. **I–K** Claudin-1, Claudin-5, and Zonula occludens-2 (ZO2) expression in hCMEC/D3 cells were decreased after treatment with LY294002. *p < 0.05 One-way ANOVA. (*n* = 3 per treatment condition). Protein expression levels were normalized to GAPDH. **L** BBB permeability measurement using the TEER assay following LY294002 or vehicle treatment in hCMEC/D3 cells. Cells were treated with media with 10 µM LY294002 or media with the vehicle at 72 h. A significant decrease in TEER was observed in the cells treated with LY294002 for up to 30 h post-treatment. ^#^*p* < 0.05 Two-way ANOVA (*n* = 7)

Although neuronal Ng expression is known to modulate the Ca^2+^-dependent pathway, recent Ng research from other cell types have reported Ca^2+^-independent regulation, including AKT signaling [22, 25, 35]. Based on our in vivo proteomic screening and ingenuity pathway analysis, we measured AKT-dependent signal transduction changes by Ng-siRNA in hCMEC/D3 cells (Fig. 5A). There was no change in total AKT expression. However, there was a significant decrease in the expression of the activated form of AKT, p-AKT (S473), and p-GSK3β, a downstream target of p-AKT. We also measured p-mTOR1 expression, another downstream target of AKT, but no change was observed. Additionally, Protein expression of VCAM1 and ICAM1, known markers of endothelial dysfunction, were also significantly increased by Ng-siRNA, indicating brain endothelial cell activation. These findings suggest that a lack of Ng in the BBB leads to decreased AKT activity, possibly contributing to Ng-mediated BBB integrity changes.

To determine the impact of Ng-mediated AKT signaling on BBB integrity, we treated hCMEC/D3 cells with two different doses of AKT inhibitor (LY294002). We measured AKT signaling, adhesion molecules, and tight junction protein expression after 24 h (Fig. 5B). Two different doses (10 mM and 25 mM) of LY294002 treatment did not alter Ng expression (Fig. 5C). As expected, LY294002 treatment significantly decreased AKT activity, as measured by p-AKT expression. A One-way ANOVA identified the statistical significance of LY294002 treatment on p-AKT expression [*F*(2,6) = 49.6, *p* < 0.0005], and Sidak’s multiple comparison tests demonstrated significance in response at both 10 and 25 mM of LY294002 treatments (Fig. 5D). Then, we measured downstream targets of AKT activation such as mTOR and GSK-3β. Similar to our Ng-siRNA-treated cells, we observed a significant decrease in GSK-3β phosphorylation following treatment of hCMEC/D3 cells with LY294002 [*F*(2,6) = 12.88, *p* < 0.01] (Fig. 5E), while there were no changes in mTOR phosphorylation (Fig. 5F). Furthermore, we quantified the expression of adhesion molecules expression, indicating endothelial dysfunction. Paradoxically we observed a decrease in the expression of ICAM1 [*F*(2,6) = 27.28, *p* < 0.001] (Fig. 5G) and VCAM1 [*F*(2,6) = 13.99, *p* < 0.01] (Fig. 5H). Notably, LY294002 treatment significantly decreases the expression of tight junction proteins, including claudin-1 [*F*(2,6) = 43, *p* < 0.0005] (Fig. 5I), claudin-5 [*F*(2,6) = 30.8, *p* < 0.0005] (Fig. 5J) and zonula occludens-2 (ZO2) [*F*(2,6) = 9.43, *p* < 0.01] (Fig. 5K) expression after 24 h. Sidak’s multiple comparison tests identified a significant effect in both 10 mM and 25 mM of LY294002 treatments. This result indicates that AKT inhibition plays an important role in tight junction protein expression in hCMEC/D3 cells.

To quantify the effect of LY294002 on the barrier integrity changes in hCMEC/D3 cells, a TEER assay was conducted in response to 10 mM of LY294002 treatments. The baseline electrical resistance of hCMEC/D3 cells was measured for 72 h. Then, the media was replaced with LY294002-containing or the vehicle-containing media and the TEER signal was monitored for the next 30 h. The 2-way ANOVA revealed that LY294002 treatment significantly decreases TEER compared to the vehicle controls in response to time [*F*(55,651) = 13.69, *p* < 0.0001] and LY294002 treatment [*F*(1,651) = 19.88, *p* < 0.0001], but no interaction [*F*(55,651) = 0.63, *p* = 0.98] (Fig. 5L). Overall, these findings indicate that Ng expression in the BBB regulates AKT activity, which impacts the expression of AKT-mediated tight junction proteins and consequently regulates BBB integrity.

## Discussion

Ng is a 7.5 kDa protein that was originally identified in neurons and is abundantly expressed in various telencephalic regions [36] [37]. Many studies have shown that Ng is concentrated in the dendrites, where it regulates postsynaptic Ca^2+^ signaling, similar to the presynaptic protein neuromodulin [38]. Ng binds to CaM at low Ca^2+^ concentrations. Ng-CaM interaction is inhibited by protein kinase C (PKC) phosphorylation of Ng. Most previous studies have highlighted that lack of Ng expression in neurons induces a detrimental effect on synaptic plasticity, synaptic regeneration, and long-term potentiation [12, 39]. Brain Ng protein expression is regulated by transcription factors such as Sp and AP2 [40], thyroid hormones [41, 42], or sleep [43], but most information is limited to the neuronal Ng expression since it was known as a neuron-specific protein. Here, we are the first to report that Ng is also expressed in brain microvasculature. Depletion of Ng expression in the BBB increases vascular permeability, mediated by a loss of AKT activation. Our findings may provide mechanistic insight into how disruptions of Ng signaling in the brain microvasculature contribute to neurological dysfunction and the pathophysiology of Alzheimer’s disease.

Increasing evidence suggests a link between disruptions in Ng expression and neurological disorders. In humans, many clinical studies have reported increased Ng levels in the cerebrospinal fluid (CSF) of Alzheimer’s disease patients [44–49]. One study correlated the increase in CSF levels of Ng with increasing cognitive decline in Alzheimer’s disease patients. Another study correlated increased CSF levels of Ng with markers of Alzheimer’s disease pathology (Aβ plaques and tau tangles) [48]. Increased CSF Ng was also reported in other neurodegenerative conditions like Parkinson’s disease and Creutzfeldt-Jakob disease (CJD) [50, 51]. Additionally, many genome-wide association studies (GWAS) have suggested that a single nucleotide polymorphism (rs12807809) located near the Ng gene is associated with schizophrenia [19, 52, 53]. Consistently, pathological studies have shown reduced Ng immunoreactivity in certain regions of the prefrontal cortex in the post-mortem brains of patients with schizophrenia [54]. Although decreased Ng has clinical relevance in human neurological diseases, the underlying mechanism remains unclear. One possible theory is that, since Ng is highly expressed in the post-synapse, decreased Ng expression may indicate the neurodegeneration of the postsynaptic neuron. However, there is no solid mechanistic link to support this theory.

The Ng knockout mouse model showed a significant decline in spatial learning and memory (Lin et al., 2023) and sensorimotor gating (Sullivan et al., 2019), consistent with findings in Alzheimer’s disease and schizophrenia patients. The loss of Ng-mediated long-term potentiation may mediate these behavioral changes [15, 55]. Ng replacement via intrahippocampal injection of a lentivirus containing a vector expressing Ng enhanced cognitive function in a mouse model for Alzheimer’s disease [56]. Overall, various studies have shown that Ng null mice exhibit hyperactivity, anxiety-like behavior, impaired learning and memory, elevated pain sensitivity, and impaired sensorimotor gating (Miyakawa et al., 2001b) [57]. These behavioral phenotypes recapitulate the behavioral symptoms of Alzheimer’s disease and schizophrenia in humans.

To date, a significant positive correlation has been established between brain Ng expression and cognitive function. A reduction in brain Ng expression has been observed in various conditions. For example, decreased expression of Ng in neurons has been observed in aging and hypothyroidism, where cognitive deficits have also been established [58, 59]. Depletion of hippocampal Ng has also been reported in vitamin A-deficient mice [60]. A loss of Ng expression and memory deficits were also noted in mice fed a high-fat diet for 20 weeks. Diet restriction in these mice was sufficient to reverse the loss of Ng expression and improve the memory deficits [61]. Interestingly, rats fed with the high-fat diet for 8 weeks demonstrated a decrease in striatal Ng expression, with only slight changes observed in the hippocampus and no changes observed in the cerebral cortex [62]. Therefore, we anticipated that a reduction in Ng expression would also negatively impact neurons and brain vasculature.

Plenty of information has recently been gathered concerning the importance of microvasculature integrity in maintaining neuronal homeostasis and function. A growing body of evidence suggests that a loss of BBB integrity may precede, exacerbate, or contribute to several neurodegenerative diseases. Disruption of the BBB has been reported in conditions such as Parkinson’s, Huntington’s, and Alzheimer’s [63]. Similarly, it is also worth noting that dysfunctions of the BBB are also reported in other neurological and psychiatric disorders [64–67]. Thus, understanding the mechanisms underlying disruptions of the BBB can lead to the development of novel therapies to manage or stall the progression of such disorders. Many studies have also reported that a loss of BBB integrity results in behavioral changes in rodents. For example, chronic stress in mice downregulates claudin-5, zonula occludens 1 (ZO1), and occludin in the nucleus accumbens (NAc). This promotes the development of depression-like behaviors in mice [68–70]. The loss of claudin-5 expression and the corresponding increase in BBB permeability also regulates schizophrenia-like behaviors in mice, such as impairments in cognitive function and sensorimotor gating [71]. Other studies showed that social isolation in female juvenile mice caused anxiety-like behaviors in adult mice, and this was linked to a decrease in claudin-5 expression and BBB breakdown in the amygdala [72]. The relationship between disruptions of the BBB and anxiety, as well as the molecular pathways that may link the two outcomes, has been described [73]. Therefore, the reduction in tight junction protein expression and increasing BBB permeability mediated by the depletion of Ng may contribute to the behavioral phenotypes observed in the Ng knockout mice.

One of the major limitations of this study is the use of conventional Ng knockout (Ng^−/−^) mice to assess in vivo BBB permeability. The Ng protein is highly expressed in excitatory neurons of the mPFC, and our study aims to investigate BBB permeability in the mPFC and its potential contribution to the known behavioral phenotypes of Ng knockout mice, including cognitive deficits with anxiety components [15, 16]. Many behavioral phenotypes associated with a loss of Ng expression are related to excitatory neurons in the mPFC. Thus, we limit the data presented in this paper to the mPFC. Although we generated endothelial-specific Ng knockout (Cre-CDH5-Ng^f/f^) mice, their behavioral phenotypes and developmental impairments remain uncharacterized. Therefore, we concentrated on the molecular effects of endothelial-specific Ng knockout using mPFC brain tissues from Cre-CDH5-Ng^f/f^ mice. We then validated the physiological impact on BBB permeability and assessed pharmacological interventions using human cerebral microvascular endothelial cells (hCMEC/D3) derived from human neocortical microvessels. Further studies employing mPFC-specific and endothelial-specific Ng knockout models are needed to elucidate the role of Ng ablation in BBB permeability and its connection to cognitive decline.

We must also consider the potential influence of neurovascular coupling, which refers to the relationship between neuronal activity and changes in cerebral blood flow. Previous studies have highlighted the critical roles that neurons and astrocytes play in BBB development and function [74–76]. For instance, neuronal Ng knockout in the mPFC could increase the demand for oxygen and nutrients, which must be supplied by the blood. To accommodate these increased needs, neurovascular coupling may enhance blood flow in the mPFC, potentially increasing BBB permeability. Consequently, Ng expression in neurons, astrocytes, and endothelial cells could influence BBB function and the neurovascular structure, but their relationship is not fully addressed by the conventional Ng knockout model used in this study. However, we observed increased apical and basolateral permeability in Ng knockout mice, suggesting elevated paracellular permeability as a result of tight junction disruption. Furthermore, our molecular findings indicate that the loss of tight junction protein expression in the mPFC of Ng knockout mice provides direct evidence of endothelial activation and increased BBB permeability rather than as a consequence of neurovascular coupling. However, future studies using neuron-specific and endothelial-specific Ng knockout models are needed to clarify the role of Ng expression in BBB structure and function.

Our brain proteomic analysis using endothelial-specific knockout mice suggested that the AKT signaling pathway may be affected by the loss of Ng in the brain vasculature. Previous studies have implicated the AKT/GSK-3β signaling pathways as essential for maintaining the BBB in physiological and pathological conditions. Granulocyte colony-stimulating factor (G-CSF), an AKT activator, stabilizes the BBB and decreases neuroinflammation in rats following neonatal hypoxia–ischemia through the inhibition of GSK-3β [77]. Moreover, treating primary brain microvascular endothelial cells differentiated from human-induced pluripotent stem (hiPS) cells with a GSK-3β inhibitor enhances barrier formation [78]. Also, inhibition of GSK-3β in brain endothelial cells promotes tight junction stability by extending the half-life of occludin and claudin-5 [79]. Since AKT inhibits GSK-3β activity by phosphorylation of the S9 residue, a loss of AKT activation leads to an increase in GSK-3β activity that may be detrimental to BBB function. Our western blot analysis of hCMEC/D3 cells revealed decreased AKT activation and subsequent GSK-3β phosphorylation in Ng-siRNA-treated cells. Interestingly, there was no change in mTOR phosphorylation, another downstream target of AKT activation. We also validated the changes in GSK-3β phosphorylation in response to AKT inhibition with LY294002, and the results demonstrated the suppression of AKT-GSK-3β signaling in the hCMEC/D3 cells, which may suppress tight junction protein expression and decrease endothelial integrity as measured by TEER. Paradoxically, AKT inhibition in hCMEC/D3 cells significantly decreases VCAM1 and ICAM1 expression, which is the opposite of what is observed in Ng knockout cells. This finding implies that AKT inhibition in the BBB impacts both endothelial activation and junctional integrity, but Ng affects VCAM1 and ICAM1 expression through an AKT-independent pathway.

Although there is no direct mechanistic link between Ng and AKT regulation, many studies have correlated a decrease in AKT activation with a decrease in Ng expression in various cell types [25, 80]. Ng expression regulates Ca^2+^-CaM complex formation, which contributes to the activation of CaMKII and calcineurin (CaN). Previous Ng studies focused on cardiomyocytes, skeletal muscle, or endothelial cells and identified the impact of Ng depletion on Ca^2+^-CaM-dependent CaN regulation. We postulate that the loss of Ng shifts Ca^2+^ dynamics in a way that activates calcineurin (CaN), leading to the dephosphorylation of AKT [81]. Our data show that there is no change in the expression of Ca^2+^-CaM-dependent proteins when Ng is depleted in brain endothelial cells; this suggests that a new Ca^2+^-CaM-independent pathway connects Ng to AKT signaling. However, further experiments will be required to confirm this hypothesis.

Our study suggests that, in addition to the neuronal consequences of a loss of Ng expression (as seen in conditions such as Alzheimer’s disease and schizophrenia), there is a significant effect of Ng expression on brain endothelial cells, causing changes in BBB permeability that may exacerbate the underlying pathological mechanisms. We have limited this study to the effects of Ng expression using conventional Ng knockout mice to establish a baseline effect of Ng expression on the BBB in the cortex. Thus, more studies are needed to evaluate the contribution of endothelial Ng depletion to the pathophysiology of these neurological and psychiatric conditions using specific disease models. These studies will not only further improve our knowledge of Ng mechanisms but may also provide novel therapeutic targets for neurological diseases through BBB integrity management.

## Supplementary Information

Below is the link to the electronic supplementary material.

Supplementary file1 (XLSX 29 KB)

## Data Availability

Proteomics data is provided within the manuscript or supplementary information files.
